# BhbZIP60 from Resurrection Plant *Boea hygrometrica* Is an mRNA Splicing-Activated Endoplasmic Reticulum Stress Regulator Involved in Drought Tolerance

**DOI:** 10.3389/fpls.2017.00245

**Published:** 2017-02-24

**Authors:** Bo Wang, Hong Du, Zhennan Zhang, Wenzhong Xu, Xin Deng

**Affiliations:** ^1^Key Laboratory of Plant Resources, Institute of Botany, Chinese Academy of SciencesBeijing, China; ^2^College of Agriculture, Xinjiang Agricultural UniversityUrumqi, China; ^3^College of Life Sciences, University of Chinese Academy of SciencesBeijing, China

**Keywords:** *Arabidopsis*, *Boea hygrometrica*, bZIP60, drought tolerance, ER-QC, mRNA-splicing, UPR

## Abstract

Adverse environmental conditions cause endoplasmic reticulum (ER) stress in plants. To mitigate ER stress damage, ER associated transcription factors and inositol-requiring enzyme-1 (IRE1)-mediated *bZIP60* mRNA splicing are activated in plants. A drought-induced gene, encoding the ortholog of AtbZIP60, was identified in the resurrection plant *Boea hygrometrica*, termed *BhbZIP60*. In response to ER stress and dehydration, *BhbZIP60* mRNA can be spliced to create a frame shift in the C terminus by the excision of 23b segment in a manner of its ortholog in other plants, thus translocating to the nucleus instead of the cytoplasm. The splicing-activated BhbZIP60 (BhbZIP60S) could function in the same way as its *Arabidopsis* ortholog by restoring the molecular phenotype of the mutant *atbzip60*. When overexpressed in *Arabidopsis, BhbZIP60S* provided transgenic plants with enhanced tolerance to drought, tunicamycin and mannitol stresses with upregulation of the expressions of ER quality control (QC) genes (*BiP2, BiP3, CNX1*, and *sPDI*) and abscisic acid (ABA) responsive genes (*RD29A, RAB18*, and *RD17*). Furthermore, in the yeast one-hybrid system, BhbZIP60S was capable of interacting with ER stress responsive elements (ERSE and ERSE-II) that exist in the promoters of known ER-QC genes, but not binding to ABA responsive *cis*-elements (ABREs). Our results demonstrated that drought-induced BhbZIP60 may have a function in drought tolerance via the splicing-activated BhbZIP60S to mediate ER-QC by direct binding to the promoters of ER-QC genes. This study evidently demonstrates the involvement of ER-QC in the drought tolerance of *Arabidopsis* and the desiccation tolerance of the resurrection plant *B. hygrometrica*.

## Introduction

Adverse environmental conditions usually cause endoplasmic reticulum (ER) stress, a phenomenon that protein folding becomes unfavorable resulting in an exceeding load of misfolded or unfolded protein accumulation in the ER (Walter and Ron, 2011; Howell, 2013). ER stress sets off the UPR, which is a homeostatic response to lighten the ER stress by bringing the protein folding and degradation capacity of the ER into alignment with the demand (Sitia and Braakman, 2003; Schröder and Kaufman, 2005). This system is defined as ER-QC, serving as an environmental sensor and responder to eliminate improperly folded proteins from the secretory pathway (Liu and Howell, 2010).

There already identified two arms of ER stress signaling pathway in plants, one involves two ER membrane-associated transcription factors (bZIP17 and bZIP28) through proteolytic cleavage, and another involves a dual protein kinase (IRE1) and its target RNA (*bZIP60*) (Howell, 2013). bZIP17 and bZIP28, the plant homologs of mammalian ATF6 (activating transcription factor 6, Schindler and Schekman, 2009), are membrane-associated transcription factors activated by various stresses in a process that involves their mobilization from the ER to the Golgi where they are processed and released by site 1 and site 2 proteases (S1P and S2P) (Liu et al., 2007a; Sun et al., 2015). Till now, AtbZIP17 (Liu et al., 2007b), ZmbZIP17 (Yang et al., 2013), AtbZIP28 (Liu et al., 2007a; Tajima et al., 2008), and OsbZIP39 (Takahashi et al., 2012) are confirmed to be activated by this way. AtbZIP60 was demonstrated to undergo an IRE1-splicing process (Deng et al., 2011). IRE1, an ER membrane-located protein kinase ribonuclease and a RNA splicing factor, has been proved to play a pivotal role for the perception of ER stress (Schröder and Kaufman, 2005; Bernales et al., 2006; Ron and Walter, 2007). The predicted structure for IRE1 splicing was based on a two “kissing” hairpin loops with conserved bases in each loop, and the predicted cleavage sites located close to the ribonuclease catalytic sites in the cytosolic domain of IRE1 (Lee et al., 2008). The splicing of the corresponding mRNA produces a frame shift resulting in a transcribed protein with a different C terminus (Yoshida et al., 2001; Deng et al., 2011). It has been reported that AtbZIP60 (Deng et al., 2011), OsbZIP50 (Hayashi et al., 2012), and ZmbZIP60 (Li et al., 2012) are activated by IRE1-catalyzed mRNA splicing, in a manner that also found in the activation of Hac1 in yeast and XBP1 in mammalian cells (Cox and Walter, 1996; Yoshida et al., 2001).

Recent studies of deficient mutants have evidently revealed the involvement of ER stress and UPR in abiotic stresses such as salt and heat. It has been found that either of the mutants in proteolytic processing, *s1p* and *bzip17*, has shown salt sensitive phenotypes (Liu et al., 2007b), while the mRNA-splicing activated *bZIP60*, showed more tolerance to salt stress when overexpressed in *Arabidopsis* (Fujita et al., 2007). This suggests that both arms of the UPR are involved in the salt stress response. It was found that heat could also activate both two arms of the UPR in *Arabidopsis*. Previous studies observed that heat treatment increases the relocation of bZIP17 and bZIP28 to the nucleus and *bzip28* single mutant has a heat sensitive phenotype (Gao et al., 2008; Che et al., 2010). Deng et al. (2011) showed that heat stress could induce the *AtbZIP60* splicing. These studies suggest that the UPR plays an important role in salt and heat tolerance.

Despite the characterization of UPR in heat and salt stresses, function of UPR in drought stress remains unknown. In the past decades, Valente et al. (2009) showed that overexpressing ER-resident molecular chaperone BiP in soybean confers resistance to drought; however, the mechanism related to ER function is still obscure. Yang et al. (2013) identified a maize membrane-bound transcription factor ZmbZIP17, which regulates target genes in both UPR and ABA-responsive pathways to mediate the crosstalk of ER-QC and ABA signaling. A global transcriptome analysis of desiccation-tolerant *Boea hygrometrica* firstly depicted that the ER-QC system might be activated to mitigate drought-induced unfolded protein stress, compared with the sensitive plants (Zhu et al., 2015). These studies indicated that UPR might play an important role in plants under drought stress, especially in the desiccation tolerant plants. However, the detailed evidence and key regulatory molecules in ER-QC are waiting decoding.

*Boea hygrometrica* is one of the widely studied dicotyledonous resurrection plants, which can tolerate extreme water loss (up to relative water content, RWC < 10%) and have the remarkable ability of being able to survive in the air-dried state for months (Jiang et al., 2007; Dinakar et al., 2012; Mitra et al., 2013). With the aid of proteomic, transcriptomic, and genomic approaches, many protective mechanisms related to photosynthesis, antioxidation, cell wall folding, and transposons have been identified in *B. hygrometrica* for adaptation to the severe drought (Jiang et al., 2007; Wang et al., 2009; Zhao et al., 2014). Similar mechanisms have also been found in other resurrection plants. For example, activation of photosynthesis and cell wall plasticity was also reported in a transcriptomic study in *Craterostigma plantagineum* antioxidant production, nitrogen remobilization, ammonia detoxification, and soluble sugar production underlying desiccation tolerance were reported in a global metabolomic analysis of *Sporobolus stapfianus* (Rodriguez et al., 2010; Bartels and Hussain, 2011; Oliver et al., 2011). Nonetheless far more mechanisms and molecules and components of the signaling pathways in desiccation tolerance need to be explored.

The previous microarray data illustrated that ER-QC-related genes including *bZIP60, bZIP49*, and *BiP2* were up-regulated during rapid desiccation in *B. hygrometrica* (Zhu et al., 2015). In this study, we cloned and identified one of the above genes, encoding a homologous protein of AtbZIP60, named *BhbZIP60*. Our results indicated that BhbZIP60 mRNA could be spliced in response to ER stress and dehydration, forming a nucleus-localized protein by frame shift. When overexpressed in *Arabidopsis*, the spliced form of BhbZIP60 is capable of binding to ER responsive elements to upregulate the UPR genes in dehydration responsiveness, thus conferring drought tolerance.

## Materials and Methods

### Plant Materials and Stress Treatments

*Boea hygrometrica* plants grown in 5 cm × 5 cm pots in a greenhouse (approximately 25°C, 16 h light/8 h dark) with regular irrigation for 3 months were used for the treatments. For dehydration treatment, fully hydrated plants were removed from the soil to Petri dishes and dried under 50% relative humidity and moderate illumination at 25°C in a climate chamber. For pharmaceutical treatments, roots of *B. hygrometrica* were submerged in 0.5 μg ml^-1^ TM, 150 mM NaCl, 20 mM MV or 100 μM ABA solutions, respectively, for indicated time. The plant materials were harvested at each time point, frozen immediately in liquid nitrogen, and stored at -80°C for RNA extraction.

The *A. thaliana* ecotype Columbia (Col-0) was used as the WT plant. *Arabidopsis thaliana* ecotype Columbia background mutant *atbzip60* (SALK_050203) was obtained from the Arabidopsis Biological Resource Center (ABRC) (Columbus, OH, USA). The surface-sterilized seeds were sown on 0.5 × MS (Murashige and Skoog, 1962) medium plates containing 1% sucrose and 0.7% agar after stratified at 4°C for 3 days in the dark. For phenotypic identification, the WT and transgenic lines seeds were prepared and grown on MS medium plus 400 mM mannitol, 2 mM DTT, or 500 μg L^-1^ TM, and the growth was evaluated and photographs were taken at the appointed time.

For soil dehydration treatments, 6-day-old seedlings were transferred from 0.5 × MS plates to soil, and grown for 14 days, and then water was withdrawn for another 14 days. The corresponding parameters were measured at that time.

### Quantification of Water Content and Actual Fluorescence Quantum Yield

Leaf water content and YII (actual fluorescence quantum yield) were monitored as describe previously (Zhao et al., 2014). Water content (%) = (fresh weight – dry weight)/fresh weight × 100. YII was measured for dark adapted leaves using PAM-101 (Walz, Germany), with a saturating light intensity of approximate 800 mmol m^-2^ s^-1^ and duration of 4.5 s. Three replicates of individual leaves were used for each treatment. For water loss of detached leaves, leaves were removed from plants that had been grown in a greening house under normal conditions. The leaves were placed on a laboratory bench and periodically weighed. The experiment was performed three times, each time with three replicate leaves per line. Water content was expressed as a percentage of fresh weight. Greening rate was measured according to the method of He et al. (2012).

### Semi-Quantitative RT-PCR (RT-PCR) and Quantitative Real-Time PCR (qRT-PCR) Analysis

Total RNA was extracted using Trizol reagent (TaKaRa). About 2 μg of total RNA, digested by DNase I (TaKaRa), was reverse transcribed into cDNA using oligo (dT) primer (TaKaRa) and MMLV Transcriptase (Promega) in a 20 μL reaction. RT-PCR was conducted by 30 cycles, with *18S* as internal controls for *Arabidopsis* and *B. hygrometrica*. qRT-PCR was performed on a Mastercycler ep realplex^2^ (Eppendorf) with *18S* as the internal controls. Quantification was performed using the 2^-ΔΔCT^ method, and the data were normalized through the quantity of the reference gene. The dissociation curves were analyzed in all amplifications. Each analysis was performed in three biological replicates.

### Molecular Cloning and Vector Construction

A contig containing *BhbZIP60* from our previous transcriptome database (Zhu et al., 2015) was found and the gene was cloned accordingly. SP were designed to amplify the sequence of *BhbZIP60* unspliced and spliced form from *B. hygrometrica* treated with DTT, using Phusion High-Fidelity DNA Polymerase (NEB, China). The sequence-confirmed PCR product was ligated into 35S promoter-driven pLeela vector according to the manufacturer’s instructions (Invitrogen). The construct was introduced into the WT and *bzip60* mutant of *Arabidopsis* by *Agrobacterium-*mediated transformation.

For determination of subcellular location, YFP-BhbZIP60U and YFP-BhbZIP60S were generated by insertion into an YFP-tag vector pENSG-YFP. The primers used in this study were listed in Supplementary Table S1. All constructs were confirmed by sequencing the entire inserted fragments.

### Sequence Alignment and Construction of Phylogenetic Tree

The RNA structure was predicted using M-fold program with default settings^1^ (Zuker, 2003). The amino acid sequences were obtained from NCBI (National Center for Biotechnology Information) database and aligned using the ClustalX 2.0 program with default settings and adjusted manually using GeneDoc software. The subcellular localization was predicted with the online software ^2^. The phylogenetic tree was constructed using the Neighbor Joining (NJ) method in Clustalx1.83 and viewed using the software MEGA6 with the following parameters: bootstrap (1,000 replicates; random seed), pair-wise deletion, and Poisson correction, according to the method of Kumar et al. (2004).

### Yeast One-Hybrid Assay

Yeast one-hybrid assays were performed according to the manufacturer’s instructions (Clontech, USA). The bait plasmids were constructed by transferring fragments containing tetramer ERSE/mERSE, ERSE-II/mERSE-II, or ABRE/mABREs elements, which were commercially synthesized and cloned into pHISi according to the method described previously (Wang et al., 2002). The full-length CDS of *BhbZIP60S* were fused to the GAL-AD in the vector *pACT2*, resulting in plasmid *pACT2-BhbZIP60S*. Each pair of bait and prey plasmids was co-transformed into yeast YM4271 cells using a lithium acetate method (Clontech, USA) and analyzed for yeast growth on selective medium containing 40 mM 3-AT but lacking histone and leucine.

### Confocal Microscopic Analysis

The plasmids containing *YFP-BhbZIP60U* or *YFP-BhbZIP60S* were transiently co-transformed with *HDEL-mCherry* into epidermal cells of *N. benthamiana* leaves via an *Agrobacterium*-mediated method (Wydro et al., 2006). Fluorescent images were obtained by laser excitation at 488 and 591 nm for YFP and mCherry, respectively. A confocal laser scanning microscope (FV1000MPE; Olympus) was used to visualize fluorescence signals. Image browser software was used for image acquisition.

## Results

### Isolation of a Drought-Induced bZIP Transcription Factor from *B. hygrometrica*

**FIGURE 1:**
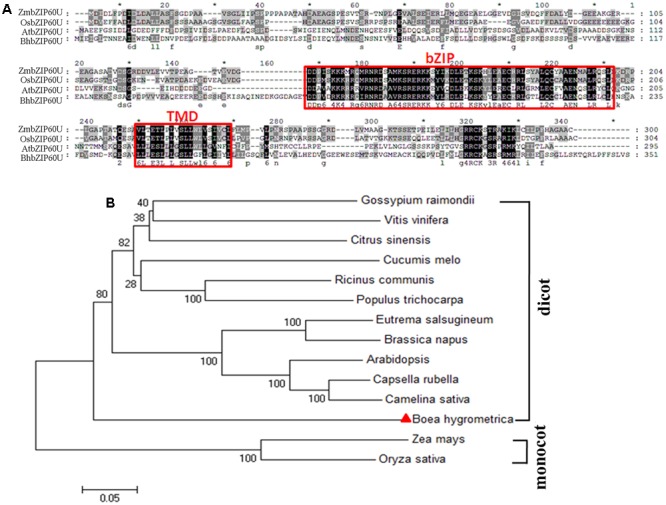
**Alignment and phylogenetic tree of bZIP60(50) from different species. (A)** The sequence alignment of unspliced form of bZIP60(50)s, from maize (ZmbZIP60U), rice (OsbZIP50U), *Arabidopsis* (AtbZIP60U), and *Boea hygrometrica* (BhbZIP60U). Sequence data (except BhbZIP60U) were obtained from the NCBI database, analyzed using ClustalX 2.0 software. The bZIP domain and TMD were indicated in red box. **(B)** The unrooted phylogenetic tree generated with ER stress-associated and membrane-associated bZIP60 factors from different plants. *Arabidopsis* (AT1G42990), rice (Os06g0622700), maize (BT086464), *Capsella rubella* (CARUB_v10009864), *Camelina sativa* (LOC104779156), *Eutrema salsugineum* (EUTSA_v10004635), *Brassica napus* (LOC106361258), *Gossypium raimondii* (LOC105770843), *Ricinus communis* (LOC8269953), *Citrus sinensis* (LOC102612558), *Populus trichocarpa* (POPTR_0005s27930), *Vitis vinifera* (LOC100244512), *Cucumis melo* (LOC103493855).

In our previous microarray analysis of the resurrection plant *B. hygrometrica* under dehydration stress, we noticed that a putative bZIP transcription factor encoding a homologous protein of AtbZIP60 was strongly induced by dehydration (Zhu et al., 2015). To better characterize its function, the full-length cDNA was cloned and designated as BhbZIP60 (**Figure 1A**). The full-length BhbZIP60 protein contains a typical bZIP domain and a TMD, which are conserved in AtbZIP60 in *Arabidopsis*, OsbZIP50 in rice, and ZmbZIP60 in maize, which are representatives of those well-studied bZIP proteins from monocotyledon and dicotyledon species (**Figure 1A**) (Deng et al., 2011; Hayashi et al., 2012; Li et al., 2012). Meanwhile, an unrooted phylogenetic tree was constructed based on the sequences of bZIP60 proteins. As shown in **Figure 1B**, BhbZIP60 belongs to the dicot branch, and is more closely related to AtbZIP60.

### *BhbZIP60* mRNA Is Spliced in *B. hygrometrica* in Response to ER Stress

**FIGURE 2:**
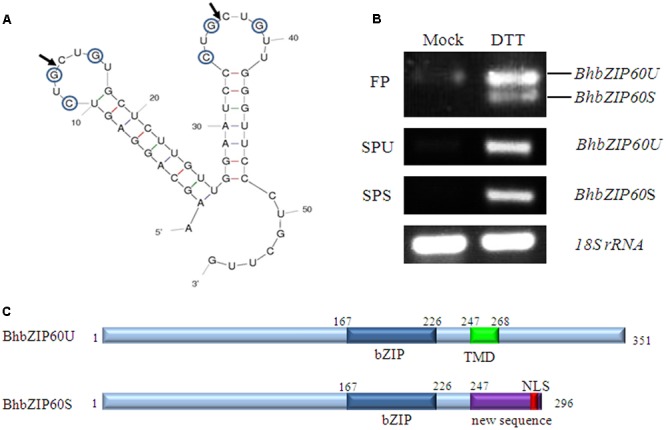
**Identification of BhbZIP60. (A)** RNA secondary structure for a segment of *BhbZIP60* mRNA, predicted using M-fold software. Circled bases represent the three conserved bases in each of the two loops. Solid block arrows indicate the predicted cleavage sites. **(B)** Electrophoresis gels of RT-PCR by splicing assay with different primers (flaking primer, FP; specific primer unspliced, SPU; specific primer spliced, SPS). RNA samples were taken from untreated or treated *B. hygrometrica* for 2 h with 2 mM DTT. **(C)** A schematic structure of BhbZIP60 protein. The predicted TMD is indicated in green. The novel sequence produced by the frameshift is indicated in purple. The NLS is indicated in red. The bZIP domain is indicated in mazarine.

To examine whether BhbZIP60 also undergo the same splicing manner as AtbZIP60, a series of studies were performed. Firstly, RNA structure was predicted with M-fold program (Zuker, 2003). The lowest free energy form of *BhbZIP60* mRNA was predicted to fold into twin kissing loops with three conserved bases in each loop, which is a potential IRE1-cutting site similar to that in *AtbZIP60* mRNA (**Figure 2A**). The expression pattern in response to ER stress was then analyzed. Three-month-old *B. hygrometrica* were exposed to ER stress agent DTT and were used to test for *BhbZIP60* splicing using RT-PCR. Two assays, a FP assay and a SP assay, were performed as described by Deng et al. (2011). The former detected both the unspliced (*BhbZIP60U*) and spliced mRNA (*BhbZIP60S*), and the latter detected either the spliced or the unspliced forms of *BhbZIP60* (Supplementary Figure 1A). The results showed that in FP assay, a single weak band was observed from untreated seedlings whereas two bands derived from the unspliced and spliced forms of *BhbZIP60* (confirmed by sequencing) were found in seedlings treated with DTT (**Figure 2B**). Meanwhile, it showed a prominent increase in both *BhbZIP60U* and *BhbZIP60S* transcripts after DTT treatment, indicating that ER stress has triggered not only the splicing of *BhbZIP60* but also its expression. The 23b-segment excision in the splicing of *BhbZIP60* RNA creates a frame shift, which resulted in the removal the existing TMD and the formation of a NLS in the new C terminus (**Figure 2C**; Supplementary Figures 1B and 2), which was similar to the counterparts in other plants (Deng et al., 2011; Hayashi et al., 2012; Li et al., 2012).

### *BhbZIP60* Expression Pattern in Response to Dehydration, ER Stress, ABA, and Other Abiotic Stresses

**FIGURE 3:**
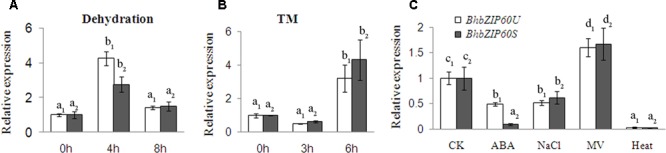
**Expression pattern of BhbZIP60. (A–C)** qRT-PCR analysis of the expression of *BhbZIP60U* and *BhbZIP60S* under dehydration **(A)**, 0.5 μg ml^-1^ TM **(B)**, and 100 μM ABA, 150 mM NaCl and 20 mM MV for 24 h, and 42°C heat shock for 1 h **(C)**. Three-month old *B. hygrometrica* were used for various treatments with *18S* rRNA gene used as the internal control. Data were expressed as the mean ± SD of three independent experiments. Different letters indicate *P* < 0.05 and the same subscripts indicate individual groups (one-way ANOVA).

Furthermore, the expression pattern of *BhbZIP60* in response to dehydration, TM (another ER stress agent, Koizumi et al., 1999), ABA, NaCl, MV, and heat shock stresses were also examined. As shown in **Figure 3A**, a sharp increase was detected in transcripts after dehydrated for 4 h, particularly in *BhbZIP60U* (**Figure 3A**). Under TM treatment, both *BhbZIP60U* and *BhbZIP60S* were induced after 6 h, with a higher level for *BhbZIP60S* (**Figure 3B**). MV, a widely used agent to induce reactive oxygen species (ROS), also triggered the transcription of both forms; however, ABA, NaCl and heat shock down-regulated the expression (**Figure 3C**). These results indicate that drought and ER stress induced transcription and produced a high splicing of *BhbZIP60* in *B. hygrometrica*, but ABA, NaCl, and heat shock showed no significant effect on the splicing of *BhbZIP60*, in comparison to *AtbZIP60* and *ZmbZIP60* under heat shock in *Arabidopsis* and maize (Deng et al., 2011; Li et al., 2012). Also, ABA-inhibited expression of *BhbZIP60* was in contrast to *ZmbZIP17*, reflecting the possibility that BhbZIP60 may not involve in the ABA signal pathway to regulated drought tolerance.

### Subcellular Localization of BhbZIP60

**FIGURE 4:**
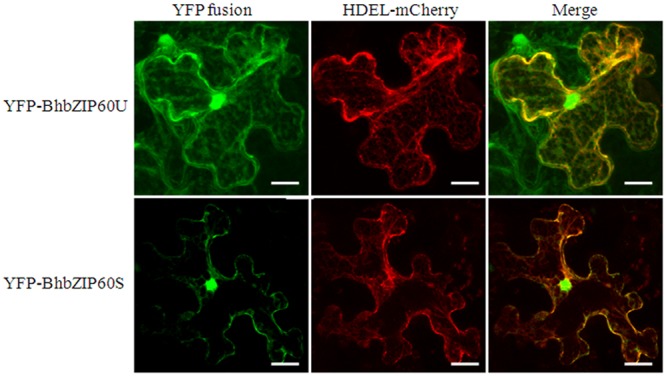
**Subcellular localization of BhbZIP60.** Subcellular localization of YFP-BhbZIP60U and YFP-BhbZIP60S were performed in a transient expression system of leaf epidermal cells in *Nicotiana benthamiana*. Green and red fluorescence represent the signal of YFP fusion protein and ER marker HDEL-mCherry, respectively. Bar = 45 μm.

The subcellular localization of BhbZIP60 was also investigated with different forms. Yellow fluorescent protein (YFP) gene-linked cDNAs representing *BhbZIP60U* and *BhbZIP60S* were transiently expressed in tobacco leaves, respectively. As expected, YFP-BhbZIP60S signal was mainly detected in nucleus (**Figure 4**, bottom), probably due to the newly formed NLS after splicing, while YFP-BhbZIP60U was co-localized in the cytoplasm with an ER marker, HDEL-mCherry, presumably from the TMD. However, apart from ER, signals were also detected in nucleus in the YFP-BhbZIP60U-expressed cells (**Figure 4**, upper). These results indicated that the mRNA-splicing processing relocated BhbZIP60 from the cytoplasm to the nucleus.

### Ectopic Expression of *BhbZIP60* Spliced Form Partially Restores Molecular Phenotype of *Arabidopsis atbzip60* Mutant

**FIGURE 5:**
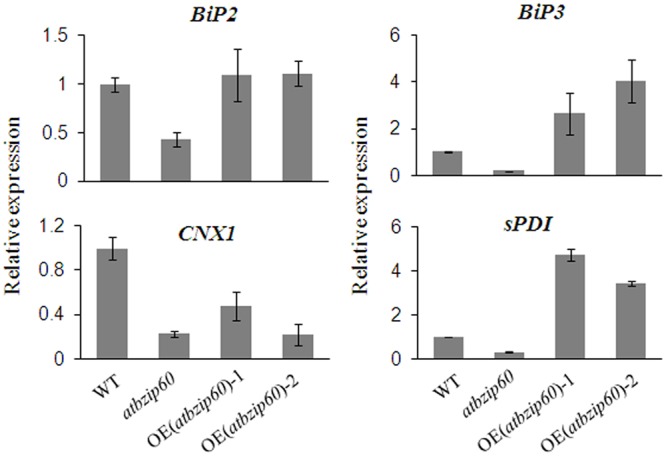
**Complementation experiment of *Arabidopsis atbzip60* mutant.** Complementation of *atbzip60* by *BhbZIP60S* was demonstrated by the expression of UPR genes detected using qRT-PCR. *OE(atbzip60)-1* and *OE(atbzip60)-2* represent overexpressing *BhbZIP60S* in *atbzip60* background. *18S* rRNA was used as an internal reference gene. Data represent the means ± SD of three independent biological replicates.

To study the molecular function of BhbZIP60, as the ortholog of *Arabidopsis* AtbZIP60, we transferred *BhbZIP60S* into *Arabidopsis atbzip60* mutant (SALK_050204), and ultimately obtained two complementary lines, *OE(atbzip60)-1* and *OE(atbzip60)-2* (Supplementary Figure 3A). In preliminary experiments, no visible difference of morphological phenotypes was observed between *atbzip60* mutant and WT with or without ABA, salt or mannitol-mediated osmotic treatments (data not shown). However, the expression of four UPR genes, *BiP2, BiP3, CNX1*, and *sPDI* was decreased in *atbzip60* mutant compared to WT plants under normal condition (**Figure 5**). Reversely, *BiP2, BiP3*, and *sPDI* displayed higher expression level in *OE(atbzip60)-1* and *OE(atbzip60)-2* than that in the mutant and WT despite that the restorage of *CNX1* expression was only observed in *OE(atbzip60)-1* (**Figure 5**). These results suggested that BhbZIP60S could substitute AtbZIP60 to regulate the UPR genes in *Arabidopsis* as illustrated by the restored molecular phenotype of *atbzip60* mutant.

### Ectopic Expression of *BhbZIP60* Spliced Form Confers Drought Tolerance in Transgenic *Arabidopsis*

**FIGURE 6:**
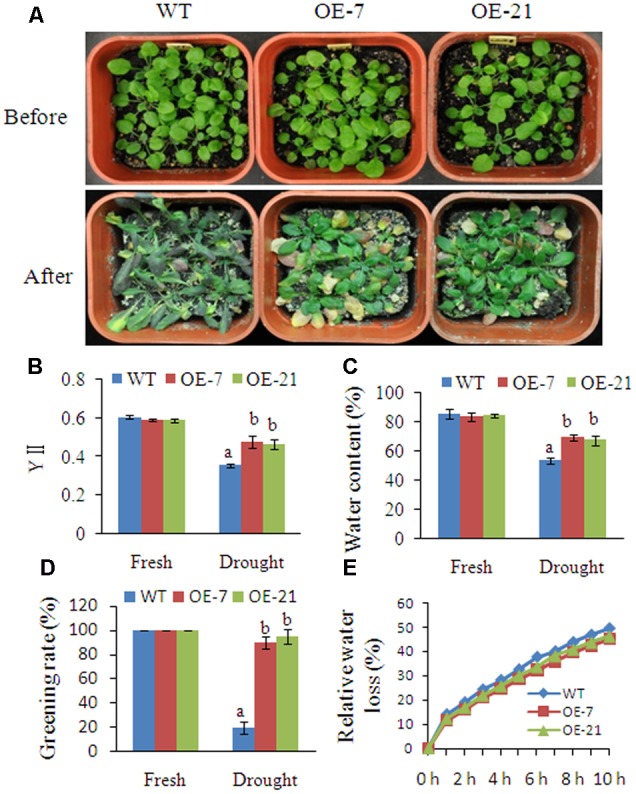
**Overexpression of *BhbZIP60S* in *Arabidopsis* confers drought tolerance. (A)** Seedlings of *BhbZIP60S* overexpression lines and WT before and after drought stress. 6-day-old seedlings were transferred from 0.5 × MS plates to soil, and grown for 14 days, and then water was withdrawn for another 14 days. **(B–D)** YII (actual fluorescence quantum yield), water content and greening rate were measured after drought stress for 14 days using unstressed plants grown in parallel as control. **(E)** Relative water loss was measured with detached leaves from seedlings grown in soil. The detached leaves were kept on the greenhouse bench, and weighed each hour for 10 h. Three independent experiments were performed. Data represent means ± SD of three independent biological replicates. Different letters indicate *P* < 0.05 (one-way ANOVA).

To investigate the physiological function of BhbZIP60, an overexpressing construct with the *BhbZIP60S* under the control of the 35S promoter was transformed into *Arabidopsis* WT Col-0. Expression of *BhbZIP60S* was detected in T3 homozygous transgenic lines (Supplementary Figure 3B). Considering the prominent desiccation tolerance of the resurrection plant *B. hygrometrica* and the dehydration-responsiveness of *BhbZIP60*, plant tolerance to severe drought tolerance was evaluated. Seedlings of *BhbZIP60S* overexpressing transgenic plants and WT were subjected to drought stress by withdrawal of water for 2 weeks. The results showed that transgenic *Arabidopsis* were found more tolerant, with higher levels of YII (actual fluorescence quantum yield, 0.46–0.47), water content (67–69%), and greening rate (90–95%) under drought condition, compared to the WT (0.35 for YII, 53% for water content and 20% for greening rate) (**Figures 6A–D**). Moreover, relative water loss with detached leaves showed that the *BhbZIP60* overexpression transgenic lines lost water more slowly than WT (**Figure 6E**). These results indicated that BhbZIP60S may function in mediating drought tolerance in *Arabidopsis*.

In comparison with WT, the transgenic plants displayed elevated green cotyledon rate, and obvious root growth with TM, and mannitol or DTT stresses, respectively (Supplementary Figure 4). However, in ABA (20 μM, 30 μM, and 50 μM) treatments, the transgenic plants did not show any obvious phenotype (Data not shown). The results suggested that BhbZIP60 also functioned in the mannitol-caused negative water potential as well as ER stress.

### Expression of *BhbZIP60S* Activates ER-QC Genes and ABA Responsive Genes

**FIGURE 7:**
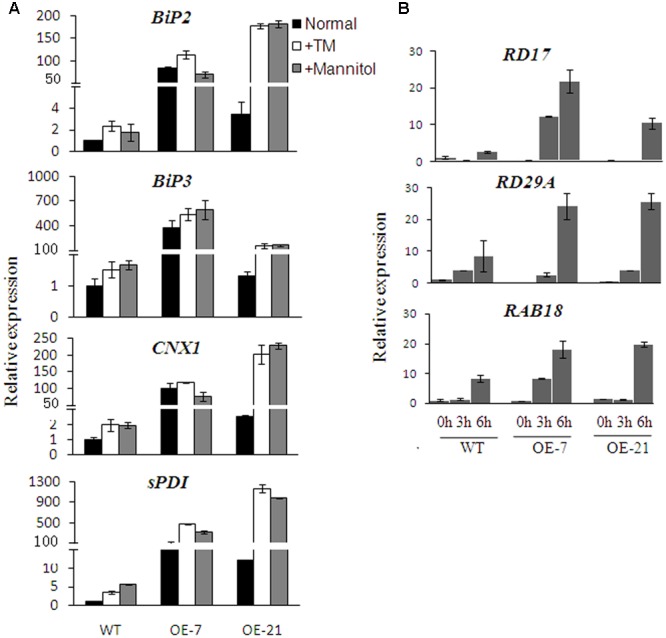
**Expression of *BhbZIP60S* activated ER-QC genes and ABA responsive genes. (A)** qRT-PCR analysis of the expression of ER-QC genes. RNA was extracted from seedlings growing on the 0.5 × MS medium with 0, 0.5 μg ml^-1^ TM or 400 mM mannitol for 3 weeks. **(B)** qRT-PCR analysis of the expression of drought-responsive genes. RNA was extracted from leaves of 4-week-old plants that were air-dried for 0 h (control), 3 h, and 6 h. Data represent means ± SD of three independent biological replicates.

To explore the internal mechanism of BhbZIP60S regulation in stress tolerance, the key genes in ER-QC, *BiP2* (bind protein), *BiP3, CNX1*, and *sPDI* (protein disulfide isomerase) were selected to characterize their expression (Anelli and Sitia, 2008). As shown in **Figure 7A**, the transcript levels of all these genes were significantly prompted in the transgenic lines compared to those in the WT, indicating that BhbZIP60S could enhance the expression of ER-QC genes under normal condition (**Figure 7A**). Under TM and mannitol treatments (mimicing the state of water deficiency), the transcript levels were much higher in the overexpressing plants than those in the WT plants (**Figure 7A**). Besides, the expression levels of the well-known ABA and drought stress-responsive genes, such as *RD29A, RD17*, and *RAB18* (Qin et al., 2008), were significantly induced in the transgenic plants compared to that in the WT plants under drought condition (**Figure 7B**). These results indicate that BhbZIP60S might regulate the response to water deficiency by mediating ER-QC through UPR signal and even triggering the drought responsive signal pathway to alleviate harm.

### BhbZIP60 Binds to ERSE and ERSE-II Elements

**FIGURE 8:**
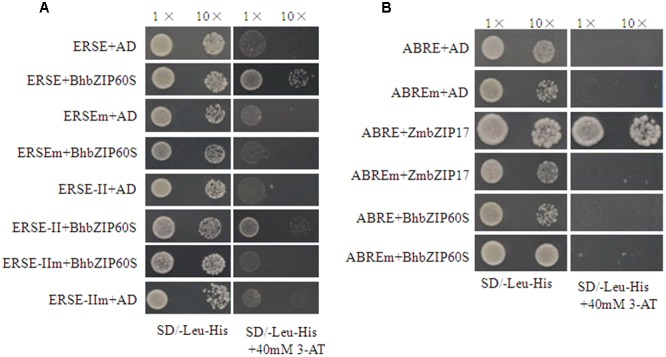
**Yeast one-hybrid analysis of the affinity of BhbZIP60S to *cis*-elements. (A)** Yeast one-hybrid analysis of the affinity of ERSE/ERSE-II and BhbZIP60S. **(B)** Yeast one-hybrid analysis of the affinity of ABRE and BhbZIP60S. Yeast cells were co-transformed with a bait vector, containing tetramer sequence fused to a HISi reported gene, and a prey vector containing the BhbZIP60S coding sequence fused to the GAL-AD. The tetramer mutant ERSEm/ERSE-IIm and ABREm were used as a negative control. ZmbZIP17 was used as a positive control. Cells were grown in liquid medium to an OD600 of 0.1 and diluted in a 10 × dilution series. Of each dilution, 5 μl was spotted on SD medium lacking leucine and histone, supplemented with 40 mM 3-AT to suppress background growth.

It is known that the promoters of *BiP2, BiP3, CNX1*, and *sPDI* contain ERSE or ERSE-II *cis*-elements, which are critical for the activation of ER stress inducible genes (Oh et al., 2003). Considering the nucleus-localized BhbZIP60S upregulated the expression of UPR genes, yeast one-hybrid assay was performed to examine the ERSE/ERSE-II-binding affinity of BhbZIP60S. The GAL4 activation domain (GAL-AD) alone was not able to bind ERSE or ERSE-II as the negative control. The result showed that BhbZIP60S could interact with ERSE/ERSE-II elements, evidenced by the growth of yeast on the media lacking leucine and histone but containing 3-AT (**Figure 8A**). In contrast, BhbZIP60S was also unable to bind to the mutated forms of ERSE or ERSE-II.

As previously mentioned, some ABA responsive genes (*RD29A, RD17*, and *RAB18*) were upregulated in the *BhbZIP60S* overexpressing plants during drought treatment (**Figure 7B**). The ABRE elements existed in the promoters of *RD29A* and *RAB18* are known critical for the activation of ABA inducible gene expression (Kang et al., 2002). A series of bZIP transcription factors that modulate ABA and stress response such as ABF/AREB/ABI5 were found to bind ABREs (Fujita et al., 2005; Yoshida et al., 2010; Liu et al., 2012). To test if BhbZIP60 regulates these ABA responsive gene by direct binding to their promoters, in a way similar to ZmbZIP17 (Yang et al., 2013), yeast one-hybrid assay was conducted and the result showed BhbZIP60S had no binding affinity to ABRE (**Figure 8B**). These results indicate that the enhanced expression of ABA responsive genes in BhbZIP60S overexpressing plants was likely via an indirect effect.

## Discussion

The image that emerges from this study is how plant could be poised to respond to environmental stress by the regulation of an elaborate mechanism-UPR signaling. UPR signaling is a positive response for plants to mitigate the ER stress. Many transcription factors across different species have been identified to play key role in UPR signaling, such as AtbZIP17/28/60 in *Arabidopsis* (Iwata and Koizumi, 2005; Liu et al., 2007a,b; Tajima et al., 2008; Deng et al., 2011), ZmbZIP17/60 in maize (Li et al., 2012; Yang et al., 2013), and OsbZIP39/50 in rice (Hayashi et al., 2012; Takahashi et al., 2012). We have shown in this study that one of the bZIP factors, BhbZIP60 from the resurrection plant *B. hygrometrica*, is subjected to mRNA-splicing, thus translocating from the ER to the nucleus when transduces ER stress signal during drought.

As a typical example of membrane-bound bZIP transcript factors, *Arabidopsis AtbZIP60* was activated via IRE1b-mediated RNA splicing in response to ER stress, in a manner previously verified by *Hac1* or *XBP1* in yeast and animal, respectively (Cox and Walter, 1996; Yoshida et al., 2001). We have also showed in this study that *BhbZIP60*, as an ortholog of *Arabidopsis bZIP60*, could undergo the similar way of mRNA-splicing to be activated under ER stress and dehydration. Firstly, the conserved IRE1-splicing structure of two “kissing” stem loops with conserved bases (**Figure 2A**); secondly, the RT-PCR and qRT-PCR assays confirmation of the two forms of *BhbZIP60* after the treatments of ER stress agents and dehydration (**Figures 2B** and **3**); thirdly, the localization of these forms changed from the cytoplasm (BhbZIP60U) to nucleus (BhbZIP60S, **Figure 4**); finally, the molecular phenotype of *Arabidopsis atbzip60* mutant has been restored by the introduction of *BhbZIP60S* (**Figure 5**). Therefore, *BhbZIP60* mRNA is able to be spliced in response to drought-induced ER stress, and then the spliced form of BhbZIP60, lacking a TMD but acquiring a putative nuclear targeting signal, presumably perform the function of transcript factor to transduce UPR signals in *B. hygrometrica* during dehydration.

From previous studies, in addition to the ER stress-induced agents (TM and DTT), abiotic stresses such as salt and heat can also induce some bZIP transcript factors from inactive to active forms through hydrolyzing the protein or splicing mRNA. As the example of undergoing proteolytic process, AtbZIP17 is activated by salt stress and then upregulates salt stress-responsive genes in *Arabidopsis* (Liu et al., 2007b); while heat stress appears to induce the proteolytic release of AtbZIP28 from the ER membrane and its null mutant has a striking heat-sensitive phenotype (Gao et al., 2008). Heat stress is also very effective in eliciting *AtbZIP60* mRNA splicing to produce active form of the transcript factor in *Arabidopsis* (Deng et al., 2011). In *B. hygrometrica*, however, we found that dehydration significantly elicited the splicing of *BhbZIP60* companied with ER stress, whereas salt and heat stresses could not (**Figure 3**). In addition to the dehydration-induced expression of *BhbZIP60*, overexpression of *BhbZIP60S* in *Arabidopsis* conferred obvious drought tolerance as well as enhanced expressions of *BiP2, BiP3, CNX1* and *sPDI* (**Figures 6** and **7**), resembling the overexpression of BiP in tobacco and soybeans (Alvim et al., 2001; Valente et al., 2009). Overexpressing *AtbZIP60* in cell suspension cultures of two different plant species rice (*Oryza sativa* L.) and white pine (*Pinus strobes* L.) also enhanced salt, drought, and cold tolerance (Tang and Page, 2013). These studies suggest that the physiological function of bZIP60 *in vivo* in response to different abiotic stresses may be due to its corresponding background plant.

Furthermore, in this study, the internal molecular mechanism of BhbZIP60-mediated drought tolerance was disclosed by the validation of the affinity of BhbZIP60 to the *cis*-elements (ERSE/ERSE-II) in the promoters (Oh et al., 2003), using yeast one-hybrid assay (**Figure 8A**). Previously, Iwata and Koizumi (2005) confirmed that the activation of UPR genes depends on the combination of AtbZIP60 and the *cis*-elements (ERSE/ERSE-II). In this study, some ABA responsive genes (*RD29A, RD17*, and *RAB18*) were also upregulated in the *Arabidopsis* plants overexpressing *BhbZIP60S*, leading to a possibility on the crosstalk of UPR and ABA-responsive signal pathway, similar to the case of ZmbZIP17 (Yang et al., 2013). However, the upregulation of these ABA responsive genes (*RD29A, RD17*, and *RAB18*) and the failure in binding of BhbZIP60 to ABREs imply an indirect effect of ABA signaling in the BhbZIP60-mediating drought tolerance (**Figures 7B** and **8B**). This is consistent to the fact that the expression of *BhbZIP60* was inhibited by ABA, which might provide a feedback inhibition mechanism to guarantee the fine tuning of BhbZIP60 (**Figure 3C**). Together these results hinted that in one hand, *BhbZIP60S* positively respond to drought stress by increasing the transcripts; in the other hand drought-induced ABA could decrease its transcripts, thus keeping the expression of *BhbZIP60* in a balance.

Thus far, we have demonstrated the mRNA-splicing activation, expression, subcellular location and molecular function of BhbZIP60 from the resurrection plant *B. hygrometrica*. These results strongly imply that BhbZIP60-mediated UPR signal under dehydration might be necessary and beneficial to the desiccation tolerance of *B. hygrometrica.*

Water deficiency usually cause the disorder of protein synthesis, degradation, folding and modifications (Dinakar et al., 2012), thus mechanisms about maintaining protein stability are particularly important. Previously, some putative osmoprotective proteins such as late embryogenesis abundant (LEA) proteins and small heat shock proteins were demonstrated to help protection of DNA, stabilization of cytoskeletal filaments and act as molecular chaperones to protect proteins, thus help resurrection plants survive desiccation (Wise and Tunnacliffe, 2004; Liu et al., 2009; Zhu et al., 2009). Interestingly, in 2015, a transcriptome analysis of *B. hygrometrica* reveals the ER-QC as one the acclimation-primed processes for the acquisition of desiccation tolerance (Zhu et al., 2015). The transcript analysis and qPCR result revealed the upregulation of some UPR genes, indicating that the protein quality control system is activated in desiccation-tolerant plants to mitigate drought-induced ER stress (Zhu et al., 2015). This is the first description on the role of ER-QC possibly involved in desiccation tolerance in resurrection plants.

In this study, *BhbZIP60* gene has been cloned and genetically characterized, leading to a deeper understanding of the mechanisms of ER-QC in desiccation tolerance in *B. hygrometrica*. In response to drought stress, BhbZIP60 translocates to the nucleus, after undergoing mRNA splicing, to bind to the *cis*-elements of UPR genes, thus mediating drought-induced ER stress. This study uncovers a new mechanism (ER-QC) to improve drought tolerance and add BhbZIP60 as a new player in the ER-QC during desiccation tolerance for the resurrection plant *B. hygrometrica.* Besides, the results also set the stage to study the role of ER-QC related processes and signals in the desiccation tolerance in resurrection plant.

## Author Contributions

XD conceived the research and experimental strategy with BW; BW performed most of the experimental works and drafted the initial manuscript under the supervision of XD; HD conducted the drought tolerance assays and the expression of ABA-responsive genes under the supervision of XD, and re-drafted the manuscript with WX; ZZ conducted the expression of UPR genes; WX and XD revised and finalized the manuscript for accuracy and concision. All authors have read and approved this manuscript.

## Conflict of Interest Statement

The authors declare that the research was conducted in the absence of any commercial or financial relationships that could be construed as a potential conflict of interest.

## Footnotes

1 http://unafold.rna.albany.edu/?q=mfold/RNA-Folding-Form

2 nls-mapper.iab.keio.ac.jp

## Abbreviations

ABA: abscisic acid
AT: aminotriazole
BhbZIP60S and BhbZIP60U: spliced and unspliced forms of BhbZIP60
BiP: binding protein
bZIP: basic domain/leucine zipper
CNX1: calnexin 1
DTT: dithiothreitol
ER-QC: endoplasmic reticulum quality control
FP: flanking primers
IRE1: inositol-requiring enzyme-1
MS: Murashige and Skoog
MV: methyl viologen
NLS: nuclear location signal
*PDI*: protein disulfide isomerase
qRT-PCR: quantitative real-time PCR
RACE: rapid-amplification of cDNA ends
RT-PCR: semi-quantitative PCR
SP: specific primers
TM: tunicamycin
TMD: transmembrane domain
UPR: unfolded protein response
WT: wild-type
