# Supplementary material for: BhbZIP60 from Resurrection Plant Boea hygrometrica Is an mRNA Splicing-Activated Endoplasmic Reticulum Stress Regulator Involved in Drought Tolerance
Source: Front Plant Sci. 2017 Feb 24;8:245. doi: 10.3389/fpls.2017.00245 (PMC5323427; doi:10.3389/fpls.2017.00245)
Supplement: Supplementary file 1 [file Data_Sheet_1.DOCX]

Supplementary Material

**BhbZIP60 from resurrection plant *Boea hygrometrica* is an mRNA splicing-activated endoplasmic reticulum stress regulator involved in drought tolerance**

**Bo Wang^1, 2, #^, Hong Du^1, 3,^ ^#^, Zhennan Zhang^1^, Wenzhong Xu^1,^ *, Xin Deng^1,^ ***

*** Correspondence:** Xin Deng: [deng@ibcas.ac.cn](mailto:deng@ibcas.ac.cn); Wenzhong Xu: [xuwzh@ibcas.ac.cn](mailto:xuwzh@ibcas.ac.cn)

## Supplementary Figures


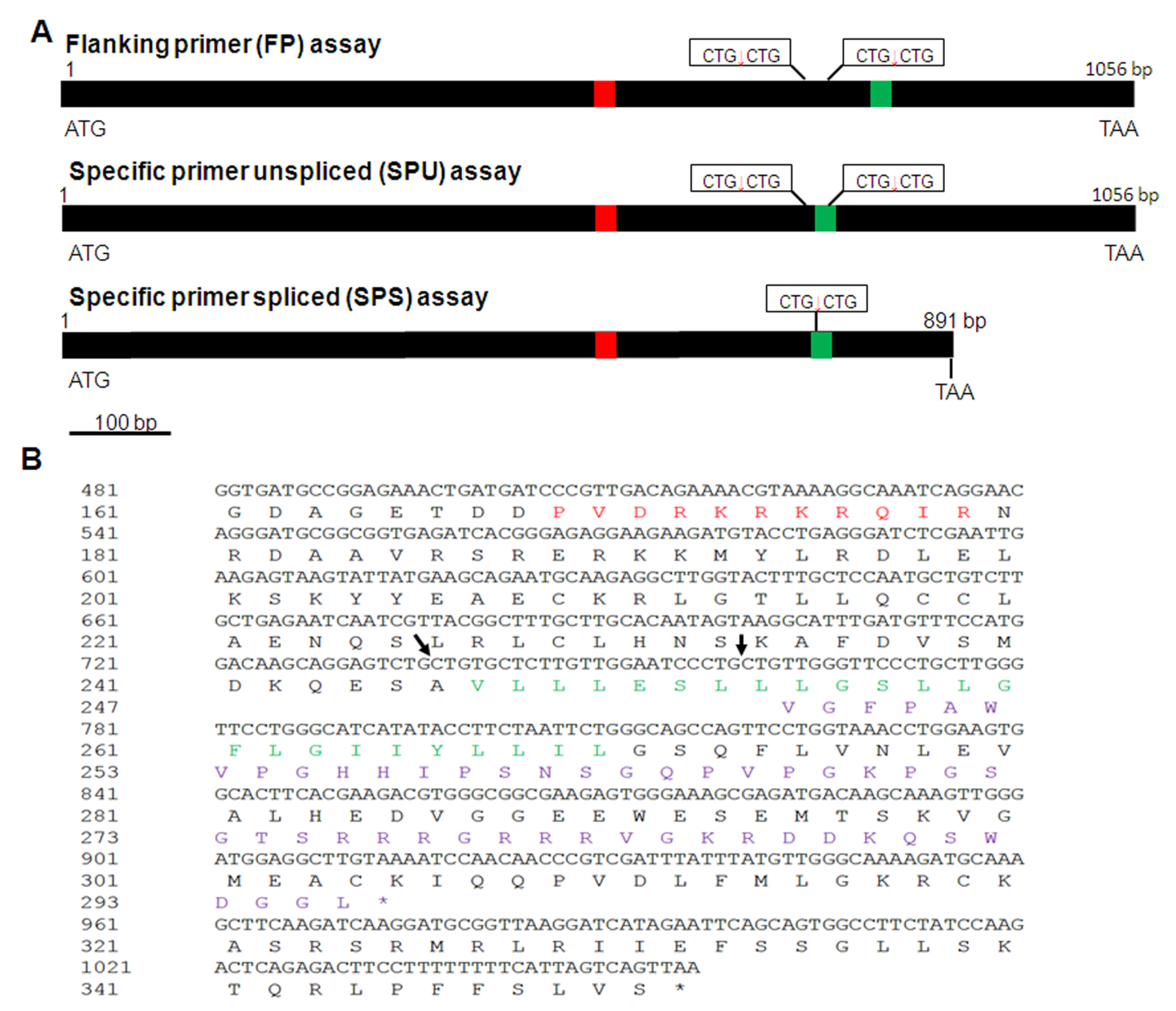


**Supplementary Figure 1. The detailed loci of the specific primers and partial sequences of** **the BhbZIP60U and BhbZIP60S.** **(A)** The detailed loci of the specific primers to amplify the *BhbZIP60U* and *BhbZIP60S* in the FP, SPU SPS assays. The red and green boxes indicated the forward primer loci and reverse primer loci, respectively. The reds arrow indicated the splicing sites. **(B)** Partial sequence of cDNA derived from unspliced and spliced forms of *BhbZIP60* mRNA. Arrows indicate splice sites inferred from the sequence of the spliced mRNA. Splice sites are indicated by arrows. The predicted TMD is indicated in green amino acids. The novel sequence produced by the frameshift is indicated in purple amino acids. *BhbZIP60U* and *BhbZIP60S* represent unspliced and spliced form of *BhbZIP60*. FP represent flanking primers assay. SPU and SPS represent specific primers unspliced and spliced assays.


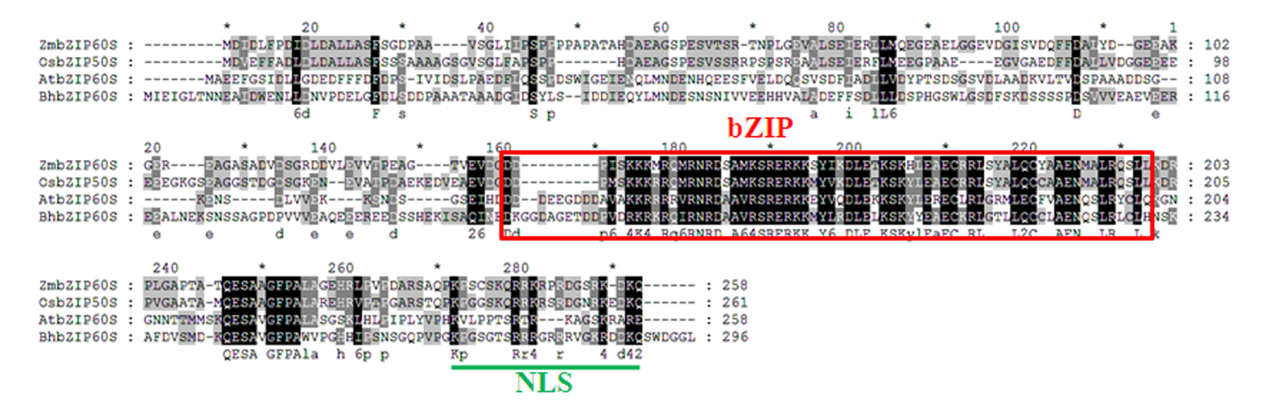


**Supplementary Figure 2.** **The sequence alignment of the spliced form of bZIP60 from *Boea hygrometrica*, Arabidopsis, rice and maize.** bZIP60S represent the spliced form. The bZIP domain is indicated in the red box. The nucleus location signal (NLS) is indicated by green underline.


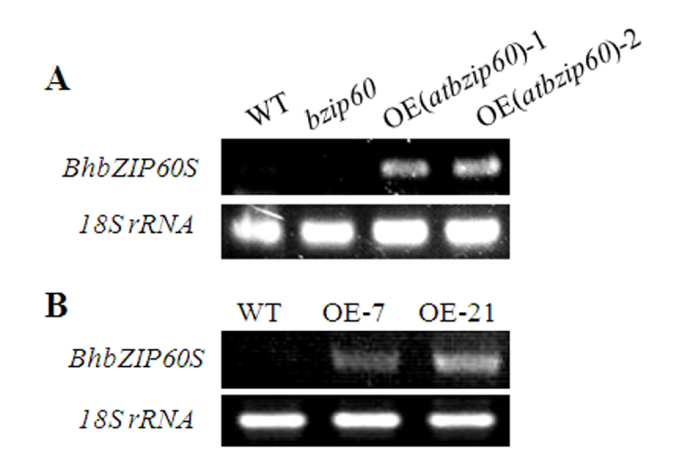


**Supplementary Figure 3. RT-PCR analysis of the expression of *BhbZIP60S* in wild-type (WT) and *atbzip60* mutant plants.**


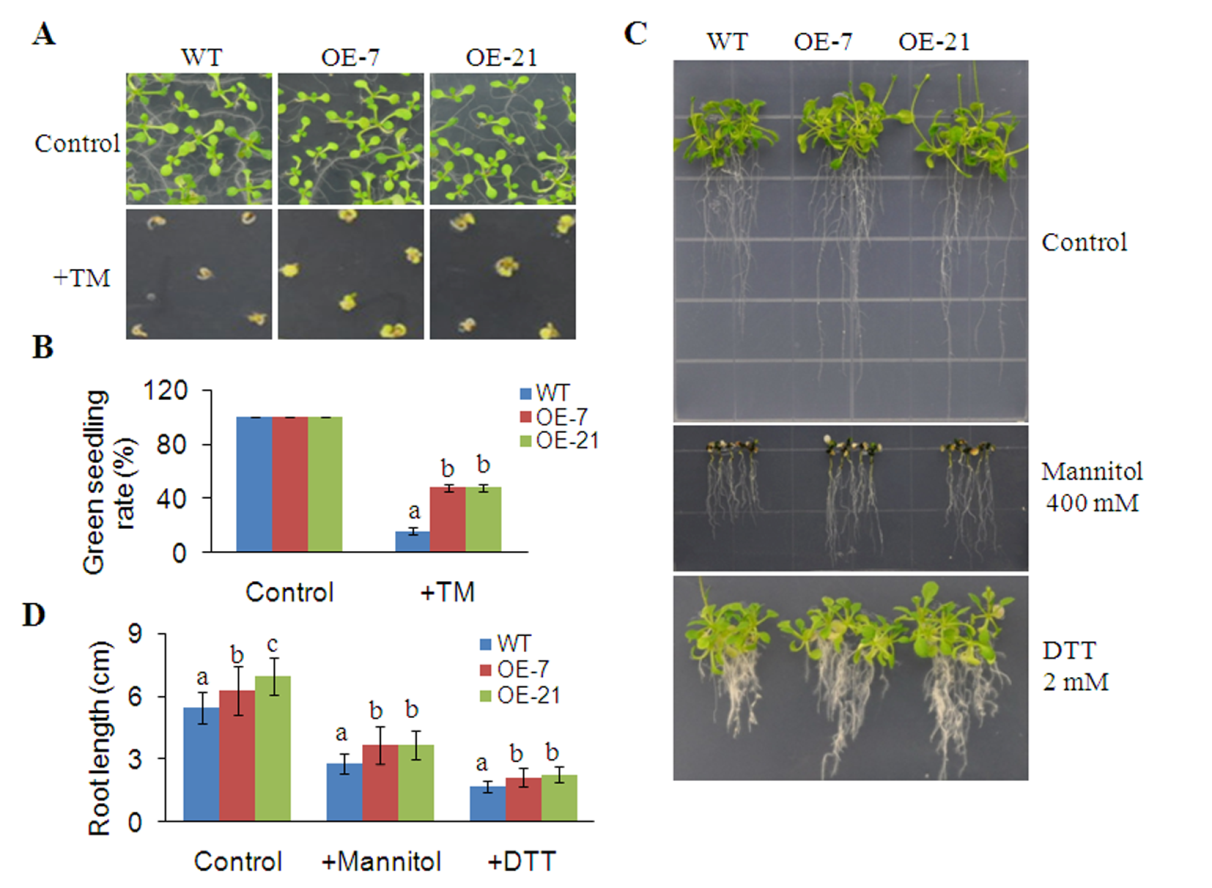


**Supplementary Figure 4. Overexpression of** ***BhbZIP60S* conferred ER stress and osmotic tolerance in Arabidopsis. (A)** Phenotype observation of wild type (WT) and *BhbZIP6*0S overexpression plants under normal and TM conditions. **(B)** Measurement of root length in **(A)**. **(C)** Phenotype observation of WT and *BhbZIP6*0S overexpression plants under normal, DTT and mannitol conditions. **(D)** Measurement of root length in **c**. Data were expressed as the mean ± SD of three independent experiments. Different letters indicate *P* < 0.05 (one-way ANOVA).

## Supplementary Table

**Table S1. Primers mentioned in this article.**

| **Gene** | **Purpose** | **Forward** | **Reverse** |
| --- | --- | --- | --- |
| BhbZIP60 (SP) | qPCR | AAGGCAAATCAGGAACAGGG | GCTGCCCAGAATTAGGAGGTAT |
| BhbZIP60 (SPU) | qPCR | AAGGCAAATCAGGAACAGGG | CAGCAGGGATTCCAACAAGAG |
| BhbZIP60 (SPS) | qPCR | AAGGCAAATCAGGAACAGGG | GGGAACCCAACAGCAGACTC |
| BiP2 | qPCR | CTTCGGATGTTTATTTGCGTT | CACCAATGAGCCTTTCAGAGT |
| BiP3 | qPCR | AGCAACCAGCACCAAGTCC | GCTTCCTCCAACAAGAACAATC |
| CNX1 | qPCR | ATGAGACAACGGCAACTATTTTCC | CCATAATCCTCATGTCCTTCACT |
| sPDI | qPCR | GCCACTAAGGCGATGATGTT | GCTCTCTGCATCACCAACAA |
| RD29A | qPCR | TGGACACGAATTCTCCATCA | TTCCAGCTCAGCTCCTGATT |
| RD17 | qPCR | ACGTCCACGCCGTTGGT | CTCCGGATGTTCCACTGGAA |
| RAB18 | qPCR | GGCTTGGGAGGAATGCTT | TTGATCTTTTGTGTTATTCCCTTCT |
| 18s rRNA | qPCR | CTTAGTTGGTGGAGCGATTTG | CCTGTTATTGCCTCAAACTTCC |
| BhbZIP60 U/S | Gene cloning | CACCATGATCGAGATTGGACT | AGGTGCTGGTTGACTGACTAATG |
| BhbZIP60 m1 | Site mutation | CTTGTTGGAATCCCTCCTGTTGGGTT | GAGGGATTCCAACAAGAGCACAGCAG |
| BhbZIP60 m2 | Site mutation | AAGCAGGAGTCTGCTTTGCTCTTGTT | AAGCAGACTCCTGCTTGTCCATGGAA |
